# Certain inequalities related with Hankel and Toeplitz determinant for *q*-starlike functions

**DOI:** 10.1016/j.heliyon.2024.e31270

**Published:** 2024-05-16

**Authors:** Ihtesham Gul, Sa'ud Al-Sa'di, Saqib Hussain, Saima Noor

**Affiliations:** a Department of Mathematics, COMSATS University Islamabad, Islamabad, 45550, Pakistan; b Government Postgraduate College Mandian, Abbottabad, 22010, Pakistan; c Department of Mathematics, Faculty of Science, The Hashemite University, P.O. Box 330127, Zarqa, 13133, Jordan; d Department of Mathematics, COMSATS University Islamabad (Abbottabad Campus), Abbottabad, 22060, Pakistan; e Department of Basic Sciences, General Administration of Preparatory Year, King Faisal University, P.O. Box 400, Al Ahsa, 31982, Saudi Arabia; f Department of Mathematics and Statistics, College of Science, King Faisal University, P.O. Box 400, Al Ahsa, 31982, Saudi Arabia

**Keywords:** 30C45, 30C50, Analytic functions, Univalent functions, Caratheodory functions, *q*-starlike functions, Hankel determinant, Toeplitz determinant, *q*-difference operator

## Abstract

In the present article, we prove the sharp upper bounds for the second order Hankel determinants $\left|H_{2}(1)|,|H_{2}(2)\right|$ and related functionals $\left|a_{2}a_{3}-a_{4}\right|$, $\left|a_{2}a_{5}-a_{3}a_{4}\right|$ for *q*-starlike functions. An upper bound for the third order Hankel determinant $\left|H_{3}(1)\right|$ along with the sharp upper bounds for Toeplitz determinant $\left|T_{m}(n)\right|$, where (m,n)∈{(2,2),(2,3),(3,1),(3,2)} are attained. Many known results are also obtained as corollaries of our main results.

## 1 Introduction

Let A represent the family of all functions *f* of the form

(1) $$f(z)=z+\sum_{n=2}^{\infty }a_{n}z^{n},$$

which are analytic in the open unit disc U={z∈C:|z|<1}. Any function *f* is said to be univalent in U if it never takes the same value twice. We denote by S the subclass of A consisting of univalent functions.

We denote by P, the class of Carathéodory functions *p* with

(2) $$p(z)=1+\sum_{n=1}^{\infty }p_{n}z^{n},$$

such that ℜ(p(z))>0 in U.

For 0≤α<1, Robertson [1] introduced the subclass $S^{⁎}(\alpha )$ of A containing all starlike functions of order *α* defined as:

A function f∈A is said to be in the class $S^{⁎}(\alpha )$ if and only if

$$\frac{1}{1-\alpha }(\frac{zf^{\prime }(z)}{f(z)}-\alpha \in P).$$

In particular, for α=0, the class $S^{⁎}(\alpha )=S^{⁎}(0)$ represents the class $S^{⁎}$ of all univalent functions which map open unit disc U onto starlike domains with respect to origin.

We also denote by C, the family of all convex functions, which map the open unit disc U onto convex domains. It is also known that a function *f* is convex if and only if $zf^{\prime }$ is starlike.

In [2], for 0<q<1, the *q*-difference operator $D_{q}:A\to A$ for f∈A is defined as follows:

(3) $$(D_{q}f)(z)=\left\{ \begin{array}{cc} \frac{f(z)-f(qz)}{z-qz}, & \text{if}\;z\neq 0,\\ f^{\prime }(0), & \text{if}\;z=0. \end{array}\right.$$

For *f* of the form (1), we have

(4) $$(D_{q}f)(z)=1+\sum_{n=2}^{\infty }{\left[n\right]}_{q}a_{n}z^{n-1},$$

where

$${\left[n\right]}_{q}=\frac{1-q^{n}}{1-q}=1+\sum_{k=1}^{n-1}q^{k},n\in N.$$

From (3) and (4) it is easy to see that

$$\lim_{q\to 1^{-}}(D_{q}f)(z)=f^{\prime }(z)$$

and

$$\lim_{q\to 1^{-}}{\left[n\right]}_{q}=n.$$

Using the q-difference operator, Ismail et al. [3] introduced the class of *q*-starlike functions as follows:

A function f∈A is said to be in the class $S_{q}^{⁎}$ if and only if

$$\left|\frac{z(D_{q}f)(z)}{f(z)}-\frac{1}{1-q}\right|\leq \frac{1}{1-q}.$$

It is important to note that when $q\to 1^{-}$, the class of *q*-starlike functions becomes the class $S^{⁎}$ of starlike functions. The notation $S_{q}^{⁎}$ for *q*-starlike functions was first used by Sahoo et al. [4].

The ${\left(m,n\right)}^{th}$ Hankel determinant of *f* is defined for m≥1,n≥1 by

$$H_{m}(n)=\left|\begin{array}{cccc} a_{n} & a_{n+1} & \ldots & a_{n+m-1}\\ a_{n+1} & a_{n+2} & \ldots & a_{n+m}\\ \vdots & \vdots & \vdots & \vdots \\ a_{n+m-1} & a_{n+m} & \ldots & a_{n+2m-2} \end{array}\right|.$$

Hankel determinants play very important role in theory of singularities and in the study of power series with integral coefficients [5], [6]. This determinant has been studied by many authors [7], [8], [9], [10] with the subject of inquiry ranging from the rate of growth of the $H_{m}(n)$ as m→∞ to the determination of the bounds with specific values of *m* and *n* for certain subfamilies of analytic functions in the open unit disc U.

For m,n∈N and functions belonging to a specific subfamily of A, finding the upper bound for $\left|H_{m}(n)\right|$ is an interesting problem to examine. A number of researchers studied the Hankel determinants $\left|H_{2}(2)|,|H_{2}(3)\right|$ and $\left|H_{3}(1)\right|$ for different subclasses of analytic functions and calculated the bounds for these functionals. Babalola [9] was the first who investigated third order hankel determinant for the classes of starlike and convex functions and he gave non-sharp upper bounds for $\left|H_{3}(1)\right|$ for these families. After then many authors contributed their findings regarding $\left|H_{m}(n)\right|$ for different values of m,n and subfamilies of analytic functions. Zaprawa [10] improved the results given in [9] by the following

(5) $$|H_{3}(1)|\leq \left\{ \begin{array}{cc} 1, & \text{if}\;\;f\in S^{⁎},\\ \frac{49}{540}, & \text{if}\;\;f\in C, \end{array}\right.$$

and asserted that these bounds are still not sharp. For the class of starlike functions the bounds obtained in (5) was further improved in 2019 by Kwon et al. [11]. In [12] Kowalczyk et al. and in [13] Lecko et al. proved the sharp bounds

$$|H_{3}(1)|\leq \frac{4}{135}\;\;\;\;\;\text{and}\;\;\;\;\;|H_{3}(1)|\leq \frac{1}{9},$$

for the classes $C\;\;\text{and}\;\;S^{⁎}(1/2)$ of convex and starlike functions of order $\frac{1}{2}$ respectively. In [14] Kowalczyk et al. solved the long-standing problem of finding the sharp upper bound

$$|H_{3}(1)|\leq \frac{4}{9},$$

for the class $S^{⁎}$ of starlike functions.

The concept of Toeplitz matrices is interrelated with Hankel matrices. In a study [15] conducted in 2015, Ye and Lim demonstrated that “Every matrix can be expressed as a product of Toeplitz matrices”, and the identical outcome holds when substituting the term “Toeplitz” with “Hankel”. The utilization of Toeplitz determinants in the context of Geometric Function Theory was initially observed in the work of Grenander and Szegö [16] in 1958. They employed Toeplitz determinants to establish the connection between first three coefficients of Carathéodory functions. Later, Thomas and Halim [17] defined the following Toeplitz determinant $T_{m}(n)$ for f∈A, represented by (1):

$$T_{m}(n)=\left|\begin{array}{cccc} a_{n} & a_{n+1} & \cdots & a_{n+m-1}\\ a_{n+1} & a_{n} & \cdots & a_{n+m-2}\\ \vdots & \vdots & \ddots & \vdots \\ a_{n+m-1} & a_{n+m-2} & \ldots & a_{n} \end{array}\right|,$$

where m≥1, n≥1 and $a_{1}=1$. In their investigation, Thomas and Halim established upper bounds on $\left|T_{m}(n)\right|$, where (m,n)∈{(2,2),(2,3),(3,1),(3,2)} and functions in $S^{⁎}$. They asserted the precision of the derived inequalities. However, a subsequent work by Ali et al. [18], provided accurate and sharp estimates, exposing a flawed assumption in the study conducted by Thomas and Halim.

Drawing inspiration from the aforementioned studies, Al-shbeil et al. [19] and Tang et al. [20] delved into the exploration of Hankel and Toeplitz determinants within specific subclasses of *q*-starlike functions. Gangania [21] explored constraints on sharp coefficient functionals for non-univalent functions. In a related context, Giri et al. [22] examined sharp initial Toeplitz determinants for certain univalent functions, while another study by the authors [23] investigated Toeplitz determinants within a class of holomorphic mappings in higher dimensions. The present article provides an extensive investigation of the Hankel and Toeplitz Determinant Problem in relation to q-starlike functions, with the objective of defining exact bounds for particular inequalities linked to these determinants. While prior studies have tackled similar inquiries within different subclasses of *q*-starlike functions, they have not comprehensively covered the entire class, and their outcomes lack sharpness. Within this article, we attain sharp upper bounds for second-order Hankel determinants $\left|H_{2}(1)|,|H_{2}(2)\right|$, and related functionals $\left|a_{2}a_{3}-a_{4}|,|a_{2}a_{5}-a_{3}a_{4}\right|$ for *q*-starlike functions. Additionally, we establish an upper bound for the third-order Hankel determinant $\left|H_{3}(1)\right|$ along with sharp upper bounds for Toeplitz determinants $\left|T_{m}(n)\right|$, where (m,n)∈{(2,2),(2,3),(3,1),(3,2)}. Furthermore, our findings yield numerous known results as corollaries of the principal outcomes. These results lay the groundwork for determining sharp upper bounds for Hankel and Toeplitz determinants of higher orders. The extremal functions identified through these inequalities have the potential to enhance previously derived inequalities for various subclasses of *q*-starlike functions.

## 2 Coefficients of functions in class $S_{q}^{⁎}$

In this section, we will find first few coefficients of the functions belonging to class $S_{q}^{⁎}$ of *q*-starlike functions and their bounds as asserted by Ismail et al. [3].

It is known [3] that if $f\in S_{q}^{⁎}$ then

(6) f(qz)=f(z)exp⁡{(ln⁡q)p(z)},

where p(z)∈P.

Using equations (2) and (6) define the function

(7) $$\phi (z)=\log(\frac{f(z)}{z})=\sum_{n=1}^{\infty }\phi_{n}z^{n},$$

we can find that

(8) $$\phi_{n}=\frac{q_{1}p_{n}}{{\left[n\right]}_{q}},$$

where

$$q_{1}=\frac{\ln q}{q-1}.$$

It can also be noted that $q_{1}\geq 1$ for 0<q<1 and $\lim_{q\to 1^{-}}q_{1}=1$.

Using equation (1) and equation (7) in the relation

$$\frac{zf^{\prime }(z)}{f(z)}=1+z\;{\left(\log(\frac{f(z)}{z})\right)}^{\prime },$$

we can write

$$\frac{z+\sum_{n=2}^{\infty }na_{n}z^{n}}{z+\sum_{n=2}^{\infty }a_{n}z^{n}}=1+z\sum_{n=1}^{\infty }n\phi_{n}z^{n-1},$$

which implies

(9) $$\sum_{n=2}^{\infty }(n-1)a_{n}z^{n}=\sum_{n=2}^{\infty }(n-1)\phi_{n-1}z^{n}+\sum_{n=3}^{\infty }(\sum_{k=3}^{n}(n-k+1)a_{k-1}\phi_{n-k+1})z^{n}.$$

Comparing coefficients of $z^{n}$ from (9) and using equation (8), we get

(10) $$a_{2}=q_{1}p_{1},$$

(11) $$a_{3}=\frac{q_{1}p_{2}}{{\left[2\right]}_{q}}+\frac{q_{1}^{2}p_{1}^{2}}{2},$$

(12) $$a_{4}=\frac{q_{1}p_{3}}{{\left[3\right]}_{q}}+\frac{q_{1}^{2}p_{1}p_{2}}{{\left[2\right]}_{q}}+\frac{q_{1}^{3}p_{1}^{3}}{6},$$

(13) $$a_{5}=\frac{q_{1}p_{4}}{{\left[4\right]}_{q}}+\frac{q_{1}^{2}p_{1}p_{3}}{{\left[3\right]}_{q}}+\frac{q_{1}^{2}p_{2}^{2}}{2{\left[2\right]}_{q}^{2}}+\frac{q_{1}^{3}p_{1}^{2}p_{2}}{2{\left[2\right]}_{q}}+\frac{q_{1}^{4}p_{1}^{4}}{24}.$$

Now we have the following result proved in [3]:

Lemma 1 *If f given by*(1)*belongs to the class*$S_{q}^{⁎}$*, then*$|a_{n}|\leq c_{n}$*with equality holds for all n if and only if*f(z)*is a rotation of*

(14) $$G_{q}(z)=z\exp(F_{q}(z))=z+\sum_{n=2}^{\infty }c_{n}z^{n}$$

*and*

(15) $$F_{q}(z)=\sum_{n=1}^{\infty }-\frac{2\ln q}{1-q^{n}}z^{n}=\sum_{n=1}^{\infty }\frac{2q_{1}}{{\left[n\right]}_{q}}z^{n}.$$

To find sharp bounds $c_{n}$, from Lemma 1, we can write:

$$\log(\frac{G_{q}(z)}{z})=F_{q}(z).$$

Differentiating with respect to *z*, we get

(16) $$\frac{zG_{q}^{\prime }(z)}{G_{q}(z)}=1+zF_{q}^{\prime }(z).$$

Using equations (14) and (15) in (16), we get

$$\frac{z+\sum_{n=2}^{\infty }nc_{n}z^{n}}{z+\sum_{n=2}^{\infty }c_{n}z^{n}}=1+2q_{1}\sum_{n=1}^{\infty }\frac{nz^{n}}{{\left[n\right]}_{q}},$$

which implies

(17) $$\sum_{n=2}^{\infty }(n-1)c_{n}z^{n}=2q_{1}\sum_{n=2}^{\infty }\frac{n-1}{{\left[n-1\right]}_{q}}z^{n}+2q_{1}\sum_{n=3}^{\infty }(\sum_{k=3}^{n}\frac{k-2}{{\left[k-2\right]}_{q}}c_{n-k+2})z^{n}.$$

Comparing coefficients of $z^{n}$ from (17), we get

(18) $$c_{2}=2q_{1},$$

(19) $$c_{3}=\frac{2q_{1}}{{\left[2\right]}_{q}}+2q_{1}^{2},$$

(20) $$c_{4}=\frac{2q_{1}}{{\left[3\right]}_{q}}+\frac{4q_{1}^{2}}{{\left[2\right]}_{q}}+\frac{4q_{1}^{3}}{3},$$

(21) $$c_{5}=\frac{2q_{1}}{{\left[4\right]}_{q}}+\frac{4q_{1}^{2}}{{\left[3\right]}_{q}}+\frac{2q_{1}^{2}}{{\left[2\right]}_{q}^{2}}+\frac{4q_{1}^{3}}{{\left[2\right]}_{q}}+\frac{2q_{1}^{4}}{3}.$$

It is interesting to note that the above values of $c_{n}$ can be obtained by using $p_{i}=2$ in the values of $a_{n}$.

## 3 A set of lemmas

Our results are also based on the following lemmas which contains the formula for $p_{2}$, the expression for $p_{3}$ and certain inequalities involving coefficients of the functions belonging to the class of Carathéodory functions.

Lemma 2 **[24]**

*If*p∈P*is given by*(2)*, then the estimates*

$$|p_{n}|\leq 2,$$

*are sharp for all*n∈N*.*

Lemma 3 **[25]**

*If*p∈P*is given by*(2)*, then for complex number μ,*

$$|p_{2}-\mu p_{1}^{2}|\leq 2\max\left\{1,|2\mu -1|\right\}.$$

Lemma 4 **[26]**

*If*p∈P*is given by*(2)*, then the estimates*

$$|p_{n}-\lambda p_{k}p_{n-k}|\leq \left\{ \begin{array}{cc} 2(2\lambda -1), & \text{if}\;\;\lambda \geq 1,\\ 2, & \text{if}\;\;0\leq \lambda \leq 1, \end{array}\right.$$

*are sharp for all*n,k∈Nandk<n*.*

Lemma 2 can also be obtained from Lemma 4 by using λ=0 and for λ=1 it gives the inequality $|p_{n}-p_{k}p_{n-k}|\leq 2$ proved by Livingston [27].

Lemma 5 *If* p∈P *is given by* (2) *, then the estimates*

$$|p_{n}-\lambda p_{k}^{2}p_{n-2k}|\leq 2+\left\{ \begin{array}{cc} 4(2\lambda -1), & \text{if}\;\;\lambda \geq 1,\\ 4, & \text{if}\;\;0<\lambda \leq 1, \end{array}\right.$$

*are sharp for all* n,k∈Nand2k<n *.*

Proof Writing $p_{n}-\lambda p_{k}^{2}p_{n-2k}=(p_{n}-p_{k}p_{n-k})+p_{k}(p_{n-k}-\lambda p_{k}p_{n-2k})$, using the triangle inequality, Lemma 2 and Lemma 4, we obtain Lemma 5. □

Lemma 6 **[16]**

*If* p∈P *is given by* (2) *, then*

$$2p_{2}=p_{1}^{2}+(4-p_{1}^{2})\zeta$$

*for some* ζ,|ζ|≤1 *and*

$$4p_{3}=p_{1}^{3}+2(4-p_{1}^{2})p_{1}\zeta -(4-p_{1}^{2})p_{1}\zeta^{2}+2(4-p_{1}^{2})(1-|\zeta {|}^{2})\eta$$

*for some* η,|η|≤1 *.*

## 4 Main results

Theorem 1 *If*$f\in S_{q}^{⁎}$*is given by*(1)*and for any complex number μ, we have*

$$|a_{3}-\mu a_{2}^{2}|\leq \frac{2q_{1}}{{\left[2\right]}_{q}}\max\left\{1,\left|(2\mu -1)q_{1}{\left[2\right]}_{q}-1\right|\right\}.$$

Proof From equations (10) and (11) we have

$$a_{3}-\mu a_{2}^{2}=(\frac{q_{1}p_{2}}{{\left[2\right]}_{q}}+\frac{q_{1}^{2}p_{1}^{2}}{2})-\mu {\left(q_{1}p_{1}\right)}^{2}=\frac{q_{1}}{{\left[2\right]}_{q}}\{p_{2}-(\mu -\frac{1}{2}){\left[2\right]}_{q}q_{1}p_{1}^{2}\}.$$

Applying Lemma 3, we get the required result. □

Corollary 1 *If*$f\in S_{q}^{⁎}$*, then the sharp inequality*

$$|H_{2}(1)|=|a_{3}-a_{2}^{2}|\leq 2q_{1}^{2}-\frac{2q_{1}}{{\left[2\right]}_{q}}$$

*holds.*

Proof Using μ=1 in Theorem 1, we get

$$|H_{2}(1)|=|a_{3}-a_{2}^{2}|\leq \frac{2q_{1}}{{\left[2\right]}_{q}}\max\left\{1,\left|q_{1}{\left[2\right]}_{q}-1\right|\right\}.$$

Now using the fact that $q_{1}{\left[2\right]}_{q}-1\geq 1$, we get the desired result. □

For $q\to 1^{-}$, we get the following well known Fekete-Szegö inequality for the class $S^{⁎}$ of starlike functions.

Corollary 2 *Let*$f\in S^{⁎}$*then*$|a_{3}-\mu a_{2}^{2}|\leq \max\left\{1,|4\mu -3|\right\}$*.*

Theorem 2 *If*$f\in S_{q}^{⁎}$*, then the sharp inequality*

$$|H_{2}(2)|=|a_{2}a_{4}-a_{3}^{2}|\leq \frac{4q_{1}^{4}}{3}+\frac{4q_{1}^{2}}{{\left[2\right]}_{q}^{2}}-\frac{4q_{1}^{2}}{{\left[3\right]}_{q}}$$

*holds.*

Proof From equations (10), (11) and (12), we have

$$H_{2}(2)=a_{2}a_{4}-a_{3}^{2}=(q_{1}p_{1})(\frac{q_{1}p_{3}}{{\left[3\right]}_{q}}+\frac{q_{1}^{2}p_{1}p_{2}}{{\left[2\right]}_{q}}+\frac{q_{1}^{3}p_{1}^{3}}{6})-{\left(\frac{q_{1}p_{2}}{{\left[2\right]}_{q}}+\frac{q_{1}^{2}p_{1}^{2}}{2}\right)}^{2}=\frac{q_{1}^{2}p_{1}p_{3}}{{\left[3\right]}_{q}}-\frac{q_{1}^{2}p_{2}^{2}}{{\left[2\right]}_{q}^{2}}-\frac{q_{1}^{4}p_{1}^{4}}{12}.$$

Using Lemma 6, we get

(22) $$H_{2}(2)=\{\frac{q_{1}^{2}p_{1}}{4{\left[3\right]}_{q}}(p_{1}^{3}+2(4-p_{1}^{2})p_{1}\zeta -(4-p_{1}^{2})p_{1}\zeta^{2}+2(4-p_{1}^{2})(1-|\zeta {|}^{2})\eta )-\frac{q_{1}^{2}}{4{\left[2\right]}_{q}^{2}}{\left(p_{1}^{2}+(4-p_{1}^{2})\zeta \right)}^{2}-\frac{q_{1}^{4}p_{1}^{4}}{12}\}=\{(\frac{q_{1}^{2}}{4{\left[3\right]}_{q}}-\frac{q_{1}^{2}}{4{\left[2\right]}_{q}^{2}}-\frac{q_{1}^{4}}{12})p_{1}^{4}+(\frac{q_{1}^{2}}{2{\left[3\right]}_{q}}-\frac{q_{1}^{2}}{2{\left[2\right]}_{q}^{2}})p_{1}^{2}(4-p_{1}^{2})\zeta -\frac{q_{1}^{2}}{4{\left[3\right]}_{q}}p_{1}^{2}(4-p_{1}^{2})\zeta^{2}-\frac{q_{1}^{2}}{4{\left[2\right]}_{q}^{2}}{\left(4-p_{1}^{2}\right)}^{2}\zeta^{2}+\frac{q_{1}^{2}}{2{\left[3\right]}_{q}}p_{1}(4-p_{1}^{2})(1-|\zeta {|}^{2})\eta \}.$$

Since the function p(z) and its rotations $p(e^{\iota \theta }z)$ where (θ∈R) are members of the class P simultaneously, we can assume without any loss of generality that $p_{1}$ is non-negative. For convenience of notation, we write

$$|p_{1}|=p_{1}=x,\;\;\text{where}\;\;x\in [0,2],|\zeta |=y,\;\;\text{where}\;\;y\in [0,1],\delta_{1}=\left|\frac{q_{1}^{2}}{4{\left[3\right]}_{q}}-\frac{q_{1}^{2}}{4{\left[2\right]}_{q}^{2}}-\frac{q_{1}^{4}}{12}\right|=\frac{q_{1}^{4}}{12}+\frac{q_{1}^{2}}{4{\left[2\right]}_{q}^{2}}-\frac{q_{1}^{2}}{4{\left[3\right]}_{q}}>0,\delta_{2}=\left|\frac{q_{1}^{2}}{2{\left[3\right]}_{q}}-\frac{q_{1}^{2}}{2{\left[2\right]}_{q}^{2}}\right|=\frac{q_{1}^{2}}{2{\left[3\right]}_{q}}-\frac{q_{1}^{2}}{2{\left[2\right]}_{q}^{2}}>0,\delta_{3}=\left|-\frac{q_{1}^{2}}{4{\left[3\right]}_{q}}\right|=\frac{q_{1}^{2}}{4{\left[3\right]}_{q}}>0,\delta_{4}=\left|-\frac{q_{1}^{2}}{4{\left[2\right]}_{q}^{2}}\right|=\frac{q_{1}^{2}}{4{\left[2\right]}_{q}^{2}}>0,\delta_{5}=\left|-\frac{q_{1}^{2}}{2{\left[3\right]}_{q}}\right|=\frac{q_{1}^{2}}{2{\left[3\right]}_{q}}>0.$$

Now applying modulus on equation (22), using the triangle inequality and |η|≤1, we have

(23) $$|H_{2}(2)|\leq \{\delta_{1}x^{4}+\delta_{2}x^{2}(4-x^{2})y+\delta_{3}x^{2}(4-x^{2})y^{2}+\delta_{4}{\left(4-x^{2}\right)}^{2}y^{2}+\delta_{5}x(4-x^{2})(1-y^{2})\}=F(x,y).$$

Next, we suppose that upper bound for (23) occurs at an interior point of the rectangle [0,2]×[0,1].

Differentiating F(x,y) partially with respect to *y*, we have:

$$\frac{\partial F}{\partial y}=(4-x^{2})((\delta_{2}+(2\delta_{3}-2\delta_{4})y)x^{2}-2\delta_{5}yx+8\delta_{4}y)$$

Now $\frac{\partial F}{\partial y}=0$ implies that either x=2 or satisfies the equation $(\delta_{2}+(2\delta_{3}-2\delta_{4})y)x^{2}-2\delta_{5}yx+8\delta_{4}y=0$. Points (x,y) satisfying such conditions are not interior points of the rectangle [0,2]×[0,1]. So that the function F(x,y) cannot have a maximum value in the interior of the rectangle and hence the maximum value shall be attained on the boundary of the rectangle.

• On the line x=0, 0≤y≤1, we have $F(0,y)=16\delta_{4}y^{2}$ and
  $$\max F(0,y)=F(0,1)=16\delta_{4}=\frac{4q_{1}^{2}}{{\left[2\right]}_{q}^{2}}.$$
• On the line x=2, 0≤y≤1, we have $F(2,y)=16\delta_{1}$ and therefore
  $$\max F(2,y)=16\delta_{1}=\frac{4q_{1}^{4}}{3}+\frac{4q_{1}^{2}}{{\left[2\right]}_{q}^{2}}-\frac{4q_{1}^{2}}{{\left[3\right]}_{q}}.$$
• On the line 0≤x≤2, y=0, we have $F(x,0)=\delta_{1}x^{4}+\delta_{5}x(4-x^{2})=g(x)$ and
  $$g^{\prime }(x)=4\delta_{1}x^{3}-3\delta_{5}x^{2}+4\delta_{5}>0,\;\;\;\;\forall \;\;\;\;0\leq x\leq 2,$$
  which shows g(x) is an increasing function on 0≤x≤2 and therefore
  $$\max F(x,0)=F(2,0)=16\delta_{1}=\frac{4q_{1}^{4}}{3}+\frac{4q_{1}^{2}}{{\left[2\right]}_{q}^{2}}-\frac{4q_{1}^{2}}{{\left[3\right]}_{q}}.$$
• On the line 0≤x≤2, y=1, we have
  $$F(x,1)=(\delta_{1}-\delta_{2}-\delta_{3}+\delta_{4})x^{4}+4(\delta_{2}+\delta_{3}-2\delta_{4})x^{2}+16\delta_{4}=h(x).$$
  Since $h^{\prime }(0)=0$ and $h^{\prime \prime }(0)=8(\delta_{2}+\delta_{3}-2\delta_{4})\leq 0$, therefore
  $$\max F(x,1)=h(0)=16\delta_{4}=\frac{4q_{1}^{2}}{{\left[2\right]}_{q}^{2}}.$$

Hence on the rectangle [0,2]×[0,1],

$$\max F(x,y)=\frac{4q_{1}^{4}}{3}+\frac{4q_{1}^{2}}{{\left[2\right]}_{q}^{2}}-\frac{4q_{1}^{2}}{{\left[3\right]}_{q}}.$$

This completes the proof. □

For $q\to 1^{-}$, we obtain the following result proved by Janteng et al. [28].

Corollary 3 *Let*$f\in S^{⁎}$*be given by*(1)*. Then the sharp inequality*

$$|a_{2}a_{4}-a_{3}^{2}|\leq 1$$

*holds.*

Theorem 3 *For* $f\in S_{q}^{⁎}$ *given by* (1) *, the sharp inequality*

$$|a_{2}a_{3}-a_{4}|\leq \frac{8q_{1}^{3}}{3}-\frac{2q_{1}}{4{\left[3\right]}_{q}}$$

*holds.*

Proof From equations (10), (11) and (12), we have

$$a_{2}a_{3}-a_{4}=(q_{1}p_{1})(\frac{q_{1}p_{2}}{{\left[2\right]}_{q}}+\frac{q_{1}^{2}p_{1}^{2}}{2})-(\frac{q_{1}p_{3}}{{\left[3\right]}_{q}}+\frac{q_{1}^{2}p_{1}p_{2}}{{\left[2\right]}_{q}}+\frac{q_{1}^{3}p_{1}^{3}}{6})=\frac{q_{1}^{3}p_{1}^{3}}{3}-\frac{q_{1}p_{3}}{{\left[3\right]}_{q}}.$$

Using Lemma 6, we get

(24) $$a_{2}a_{3}-a_{4}=\frac{q_{1}^{3}p_{1}^{3}}{3}-\frac{q_{1}}{4{\left[3\right]}_{q}}(p_{1}^{3}+2(4-p_{1}^{2})p_{1}\zeta -(4-p_{1}^{2})p_{1}\zeta^{2}+2(4-p_{1}^{2})(1-|\zeta {|}^{2})\eta )=\{(\frac{q_{1}^{3}}{3}-\frac{q_{1}}{4{\left[3\right]}_{q}})p_{1}^{3}-\frac{q_{1}}{2{\left[3\right]}_{q}}(4-p_{1}^{2})p_{1}\zeta +\frac{q_{1}}{4{\left[3\right]}_{q}}(4-p_{1}^{2})p_{1}\zeta^{2}-\frac{q_{1}}{2{\left[3\right]}_{q}}(4-p_{1}^{2})(1-|\zeta {|}^{2})\eta \}.$$

We can assume $p_{1}$ is non-negative and for convenience of notation, we write

$$|p_{1}|=p_{1}=x,\;\;\text{where}\;\;x\in [0,2],|\zeta |=y,\;\;\text{where}\;\;y\in [0,1],\sigma_{1}=\left|\frac{q_{1}^{3}}{3}-\frac{q_{1}}{4{\left[3\right]}_{q}}\right|=\frac{q_{1}^{3}}{3}-\frac{q_{1}}{4{\left[3\right]}_{q}}>0,\sigma_{2}=\left|-\frac{q_{1}}{4{\left[3\right]}_{q}}\right|=\frac{q_{1}}{4{\left[3\right]}_{q}}>0.$$

Applying modulus on equation (24), using the triangle inequality and |η|≤1, we have

(25) $$|a_{2}a_{3}-a_{4}|\leq \sigma_{1}x^{3}+2\sigma_{2}(4-x^{2})xy+\sigma_{2}(4-x^{2})xy^{2}+2\sigma_{2}(4-x^{2})(1-y^{2})=G(x,y).$$

Next, we assume that upper bound for (25) occurs at an interior point of the rectangle [0,2]×[0,1].

Differentiating G(x,y) partially with respect to *y*, we have:

$$\frac{\partial G}{\partial y}=2\sigma_{2}(4-x^{2})(x+xy-2y).$$

Now $\frac{\partial G}{\partial y}=0$ implies that either x=2 or $x=\frac{2y}{1+y}$. Points (x,y) satisfying such conditions are not interior points of the rectangle [0,2]×[0,1]. So that the function G(x,y) cannot have a maximum value in the interior of the rectangle and hence the maximum value shall be attained on the boundary of the rectangle.

• On the line x=0, 0≤y≤1, we have $G(0,y)=2\sigma_{2}(4-0^{2})(1-y^{2})=8\sigma_{2}(1-y^{2})$ and
  $$\max G(0,y)=G(0,0)=8\sigma_{2}=\frac{2q_{1}}{{\left[3\right]}_{q}}.$$
• On the line x=2, 0≤y≤1, we have $G(2,y)=8\sigma_{1}$ and therefore
  $$\max G(2,y)=8\sigma_{1}=\frac{8q_{1}^{3}}{3}-\frac{2q_{1}}{{\left[3\right]}_{q}}.$$
• On the line 0≤x≤2, y=0, we have $G(x,0)=\sigma_{1}x^{3}+2\sigma_{2}(4-x^{2})=k(x)$.
  Since $k^{\prime }(0)=0$ and $k^{\prime \prime }(0)=-4\sigma_{2}<0$, therefore
  $$\max G(x,0)=k(0)=8\sigma_{2}=\frac{2q_{1}}{{\left[3\right]}_{q}}.$$
• On the line 0≤x≤2, y=1, we have $G(x,1)=\sigma_{1}x^{3}+3\sigma_{2}(4-x^{2})x=s(x)$.
  Since $s^{\prime }(x)=3\sigma_{1}x^{2}+12\sigma_{2}-9\sigma_{2}x^{2}=(3\sigma_{1}-9\sigma_{2})x^{2}+12\sigma_{2}\geq 0$, therefore s(x) is an increasing function on [0,2] and
  $$\max G(x,1)=s(2))=8\sigma_{1}=\frac{8q_{1}^{3}}{3}-\frac{2q_{1}}{{\left[3\right]}_{q}}.$$

Therefore on the rectangle [0,2]×[0,1],

$$\max G(x,y)=\frac{8q_{1}^{3}}{3}-\frac{2q_{1}}{{\left[3\right]}_{q}}.$$

Hence the result follows. □

Theorem 4 *For*$f\in S_{q}^{⁎}$*given by*(1)*, the sharp inequality*

$$|a_{2}a_{5}-a_{3}a_{4}|\leq \frac{4q_{1}^{5}}{3}+\frac{8q_{1}^{4}}{3{\left[2\right]}_{q}}-\frac{4q_{1}^{3}}{{\left[3\right]}_{q}}+\frac{4q_{1}^{3}}{{\left[2\right]}_{q}^{2}}-\frac{4q_{1}^{2}}{{\left[4\right]}_{q}}+\frac{4q_{1}^{2}}{{\left[2\right]}_{q}{\left[3\right]}_{q}}$$

*holds.*

Proof From equations (10), (11), (12) and (13), we have

$$a_{2}a_{5}-a_{3}a_{4}=\{(q_{1}p_{1})(\frac{q_{1}p_{4}}{{\left[4\right]}_{q}}+\frac{q_{1}^{2}p_{1}p_{3}}{{\left[3\right]}_{q}}+\frac{q_{1}^{2}p_{2}^{2}}{2{\left[2\right]}_{q}^{2}}+\frac{q_{1}^{3}p_{1}^{2}p_{2}}{2{\left[2\right]}_{q}}+\frac{q_{1}^{4}p_{1}^{4}}{24})-(\frac{q_{1}p_{2}}{{\left[2\right]}_{q}}+\frac{q_{1}^{2}p_{1}^{2}}{2})(\frac{q_{1}p_{3}}{{\left[3\right]}_{q}}+\frac{q_{1}^{2}p_{1}p_{2}}{{\left[2\right]}_{q}}+\frac{q_{1}^{3}p_{1}^{3}}{6})\}=-\frac{q_{1}^{5}p_{1}^{5}}{24}-\frac{q_{1}^{4}p_{1}^{3}p_{2}}{6{\left[2\right]}_{q}}+\frac{q_{1}^{3}p_{1}^{2}p_{3}}{2{\left[3\right]}_{q}}-\frac{q_{1}^{3}p_{1}p_{2}^{2}}{2{\left[2\right]}_{q}^{2}}+\frac{q_{1}^{2}p_{1}p_{4}}{{\left[4\right]}_{q}}-\frac{q_{1}^{2}p_{2}p_{3}}{{\left[2\right]}_{q}{\left[3\right]}_{q}}=-\frac{1}{24}\{\frac{6q_{1}^{2}}{{\left[2\right]}_{q}{\left[3\right]}_{q}}(\frac{q_{1}{\left[2\right]}_{q}}{2}p_{1}^{2}-p_{2})(\frac{q_{1}^{2}{\left[3\right]}_{q}}{3}p_{1}^{3}-p_{3})\;\;+\frac{18q_{1}^{2}}{{\left[2\right]}_{q}{\left[3\right]}_{q}}(\frac{q_{1}{\left[2\right]}_{q}}{2}p_{1}^{2}-p_{2})(\frac{2q_{1}{\left[3\right]}_{q}}{3{\left[2\right]}_{q}}p_{1}p_{2}-p_{3})+\frac{24q_{1}^{2}p_{1}}{{\left[4\right]}_{q}}(\frac{q_{1}{\left[4\right]}_{q}}{{\left[2\right]}_{q}^{2}}p_{2}^{2}-p_{4})\}.$$

Applying the triangle inequality and using Lemma 2, Lemma 4 and Lemma 5, we have:

$$|a_{2}a_{5}-a_{3}a_{4}|\leq \frac{q_{1}^{2}}{{\left[2\right]}_{q}{\left[3\right]}_{q}}(q_{1}{\left[2\right]}_{q}-1)(\frac{4q_{1}^{2}{\left[3\right]}_{q}}{3}-1)+\frac{3q_{1}^{2}}{{\left[2\right]}_{q}{\left[3\right]}_{q}}(q_{1}{\left[2\right]}_{q}-1)(\frac{4q_{1}{\left[3\right]}_{q}}{3{\left[2\right]}_{q}}-1)+\frac{4q_{1}^{2}}{{\left[4\right]}_{q}}(\frac{2q_{1}{\left[4\right]}_{q}}{{\left[2\right]}_{q}^{2}}-1)=\frac{4q_{1}^{5}}{3}+\frac{8q_{1}^{4}}{3{\left[2\right]}_{q}}-\frac{4q_{1}^{3}}{{\left[3\right]}_{q}}+\frac{4q_{1}^{3}}{{\left[2\right]}_{q}^{2}}-\frac{4q_{1}^{2}}{{\left[4\right]}_{q}}+\frac{4q_{1}^{2}}{{\left[2\right]}_{q}{\left[3\right]}_{q}}.$$

Hence the result follows. □

For $q\to 1^{-}$, we obtain the following result proved by Zaprawa [10].

Corollary 4 *Let*$f\in S^{⁎}$*then*

$$|a_{2}a_{5}-a_{3}a_{4}|\leq 2.$$

Remark 1 All the inequalities obtained in Theorem 1, Theorem 2, Theorem 3 and Theorem 4 are sharp for the rotations of the function $G_{q}(z)$ given in equation (14).

The following result is not sharp:

Theorem 5 *For*$f\in S_{q}^{⁎}$*given by*(1)*,*

$$|H_{3}(1)|=|(a_{3}a_{5}-a_{4}^{2})+a_{2}(a_{4}a_{3}-a_{5}a_{2})+a_{3}(a_{2}a_{4}-a_{3}^{2})|\leq B_{1}(q),$$

*where*

(26) $$B_{1}(q)=\frac{1}{9}\{4q_{1}^{6}-\frac{12}{{\left[2\right]}_{q}}q_{1}^{5}+(\frac{116}{{\left[2\right]}_{q}^{2}}+\frac{24}{{\left[3\right]}_{q}})q_{1}^{4}-(\frac{36}{{\left[4\right]}_{q}}+\frac{44}{{\left[2\right]}_{q}^{3}}+\frac{120}{{\left[2\right]}_{q}{\left[3\right]}_{q}})q_{1}^{3}+(\frac{36}{{\left[2\right]}_{q}{\left[4\right]}_{q}}+\frac{36}{{\left[3\right]}_{q}^{2}})q_{1}^{2}\}.$$

Proof From equations (10), (11), (12) and (13), we have

$$(a_{3}a_{5}-a_{4}^{2})+a_{2}(a_{4}a_{3}-a_{5}a_{2})+a_{3}(a_{2}a_{4}-a_{3}^{2})=\{\frac{q_{1}^{2}p_{2}p_{4}}{{\left[2\right]}_{q}{\left[4\right]}_{q}}-\frac{q_{1}^{2}p_{3}^{2}}{{\left[3\right]}_{q}^{2}}-\frac{q_{1}^{3}p_{2}^{3}}{2{\left[2\right]}_{q}^{3}}+\frac{q_{1}^{3}p_{1}p_{2}p_{3}}{{\left[2\right]}_{q}{\left[3\right]}_{q}}-\frac{q_{1}^{3}p_{1}^{2}p_{4}}{2{\left[4\right]}_{q}}-\frac{q_{1}^{4}p_{1}^{2}p_{2}^{2}}{4{\left[2\right]}_{q}^{2}}+\frac{q_{1}^{4}p_{1}^{3}p_{3}}{6{\left[3\right]}_{q}}+\frac{q_{1}^{5}p_{1}^{4}p_{2}}{24{\left[2\right]}_{q}}-\frac{q_{1}^{6}p_{1}^{6}}{144}\}=\frac{1}{18}\{\frac{10q_{1}^{2}}{{\left[2\right]}_{q}{\left[4\right]}_{q}}(p_{2}-\frac{q_{1}{\left[2\right]}_{q}}{2}p_{1}^{2})(p_{4}-\frac{q_{1}[4_{q}]}{{\left[2\right]}_{q}^{2}}p_{2}^{2})+\frac{8q_{1}^{2}}{{\left[2\right]}_{q}{\left[4\right]}_{q}}(p_{2}-\frac{q_{1}{\left[2\right]}_{q}}{2}p_{1}^{2})(p_{4}-\frac{3q_{1}{\left[4\right]}_{q}}{4{\left[3\right]}_{q}}p_{1}p_{3})+\frac{q_{1}^{3}}{{\left[2\right]}_{q}^{3}}{\left(p_{2}-\frac{q_{1}{\left[2\right]}_{q}}{2}p_{1}^{2}\right)}^{3}-\frac{18q_{1}^{2}}{{\left[3\right]}_{q}^{2}}{\left(p_{3}-\frac{2q_{1}{\left[3\right]}_{q}}{3{\left[2\right]}_{q}}p_{1}p_{2}\right)}^{2}\}.$$

Using the triangle inequality and Lemma 4, we have

$$|H_{3}(1)|\leq \frac{1}{9}\{\frac{20q_{1}^{2}}{{\left[2\right]}_{q}{\left[4\right]}_{q}}(q_{1}{\left[2\right]}_{q}-1)(\frac{2q_{1}{\left[4\right]}_{q}}{{\left[2\right]}_{q}^{2}}-1)+\frac{16q_{1}^{2}}{{\left[2\right]}_{q}{\left[4\right]}_{q}}(q_{1}{\left[2\right]}_{q}-1)\;(\frac{3q_{1}{\left[4\right]}_{q}}{2{\left[3\right]}_{q}}-1)+\frac{4q_{1}^{3}}{{\left[2\right]}_{q}^{3}}{\left(q_{1}{\left[2\right]}_{q}-1\right)}^{3}+\frac{36q_{1}^{2}}{{\left[3\right]}_{q}^{2}}{\left(\frac{4q_{1}{\left[3\right]}_{q}}{3{\left[2\right]}_{q}}-1\right)}^{2}\}\leq \frac{1}{9}\{\frac{40q_{1}^{4}}{{\left[2\right]}_{q}^{2}}+\frac{24q_{1}^{4}}{{\left[3\right]}_{q}}-\frac{36q_{1}^{3}}{{\left[4\right]}_{q}}-\frac{40q_{1}^{3}}{{\left[2\right]}_{q}^{3}}-\frac{24q_{1}^{3}}{{\left[2\right]}_{q}{\left[3\right]}_{q}}+\frac{36q_{1}^{2}}{{\left[2\right]}_{q}{\left[4\right]}_{q}}+4q_{1}^{6}-\frac{4q_{1}^{3}}{{\left[2\right]}_{q}^{3}}-\frac{12q_{1}^{5}}{{\left[2\right]}_{q}}+\frac{12q_{1}^{4}}{{\left[2\right]}_{q}^{2}}+\frac{64q_{1}^{4}}{{\left[2\right]}_{q}^{2}}+\frac{36q_{1}^{2}}{{\left[3\right]}_{q}^{2}}-\frac{96q_{1}^{3}}{{\left[2\right]}_{q}{\left[3\right]}_{q}}\}=\frac{1}{9}\{4q_{1}^{6}-\frac{12}{{\left[2\right]}_{q}}q_{1}^{5}+(\frac{116}{{\left[2\right]}_{q}^{2}}+\frac{24}{{\left[3\right]}_{q}})q_{1}^{4}-(\frac{36}{{\left[4\right]}_{q}}+\frac{44}{{\left[2\right]}_{q}^{3}}+\frac{120}{{\left[2\right]}_{q}{\left[3\right]}_{q}})q_{1}^{3}+(\frac{36}{{\left[2\right]}_{q}{\left[4\right]}_{q}}+\frac{36}{{\left[3\right]}_{q}^{2}})q_{1}^{2}\}.$$

As required. □

For $q\to 1^{-}$, we have the following result proved by Zaprawa [10].

Corollary 5 *For*$f\in S^{⁎}$*,*

$$|H_{3}(1)|=|(a_{3}a_{5}-a_{4}^{2})+a_{2}(a_{4}a_{3}-a_{5}a_{2})+a_{3}(a_{2}a_{4}-a_{3}^{2})|\leq 1.$$

Remark 2 For $f\in S_{q}^{⁎}$, an upper bound

(27) $$B_{2}(q)=\{\frac{44}{9}q_{1}^{6}+\frac{52}{3{\left[2\right]}_{q}}q_{1}^{5}+(\frac{32}{3{\left[3\right]}_{q}}+\frac{4}{{\left[2\right]}_{q}^{2}})q_{1}^{4}-(\frac{4}{{\left[2\right]}_{q}^{3}}+\frac{4}{{\left[4\right]}_{q}}-\frac{16}{{\left[2\right]}_{q}{\left[3\right]}_{q}})q_{1}^{3}-(\frac{4}{{\left[3\right]}_{q}^{2}}+\frac{4}{{\left[2\right]}_{q}{\left[4\right]}_{q}})q_{1}^{2}\}$$

for $\left|H_{3}(1)\right|$ can also be obtained from the inequality

$$\left|H_{3}(1)|\leq |a_{3}||a_{2}a_{4}-a_{3}^{2}|+|a_{4}||a_{2}a_{3}-a_{4}|+|a_{5}||a_{3}-a_{2}^{2}\right|$$

by using Theorem 1, Theorem 2, Theorem 3 and Lemma 1 but this bound is very large as compared to that in (26). Few examples are given in the following table

**Table:** 

*q*	*B*_2_(*q*)	*B*_2_(*q*)
0.1	289.0972	3717.7884
0.5	7.6508	125.0988
0.7	2.9386	51.2340
0.9	1.3853	25.0557
0.99	1.0319	18.8939

. From (27), when $q\to 1^{-}$, one can also obtain the following result proved by Babalola [9].

Corollary 6 *For* $f\in S^{⁎}$ *,*

$$|H_{3}(1)|=|(a_{3}a_{5}-a_{4}^{2})+a_{2}(a_{4}a_{3}-a_{5}a_{2})+a_{3}(a_{2}a_{4}-a_{3}^{2})|\leq 16.$$

Theorem 6 *If*$f\in S_{q}^{⁎}$*, then*

(28) $$|T_{2}(1)|=|a_{1}^{2}-a_{2}^{2}|\leq 1+4q_{1}^{2},$$

(29) $$|T_{2}(2)|=|a_{2}^{2}-a_{3}^{2}|\leq 4q_{1}^{2}+\frac{4q_{1}^{2}}{{\left[2\right]}_{q}^{2}}+\frac{8q_{1}^{3}}{{\left[2\right]}_{q}}+4q_{1}^{4},$$

(30) $$|T_{2}(3)|=|a_{3}^{2}-a_{4}^{2}|\leq \{\frac{4q_{1}^{2}}{{\left[2\right]}_{q}^{2}}+\frac{4q_{1}^{2}}{{\left[3\right]}_{q}^{2}}+\frac{8q_{1}^{3}}{{\left[2\right]}_{q}}+\frac{16q_{1}^{3}}{{\left[2\right]}_{q}{\left[3\right]}_{q}}+4q_{1}^{4}+\frac{16q_{1}^{4}}{{\left[2\right]}_{q}^{2}}+\frac{16q_{1}^{4}}{3{\left[3\right]}_{q}}+\frac{32q_{1}^{5}}{3{\left[2\right]}_{q}}+\frac{16q_{1}^{6}}{9}\}.$$

*All the inequalities are sharp.*

Proof Let $f\in S_{q}^{⁎}$ be given by equation (1), then

$$T_{2}(n)=\left|\begin{array}{cc} a_{n} & a_{n+1}\\ a_{n+1} & a_{n} \end{array}\right|=a_{n}^{2}-a_{n+1}^{2}.$$

Taking absolute and applying the triangle inequality, we have

$$|T_{2}(n)|=|a_{n}^{2}-a_{n+1}^{2}|\leq |a_{n}^{2}|+|a_{n+1}^{2}|.$$

Putting n=1,n=2,n=3 and using Lemma 1, we attain the required inequalities.

To prove the sharpness consider the function

(31) $$E_{q}(z)=z\exp(F_{q}(iz)),$$

where $F_{q}(z)$ is defined in (15). By following a similar mathematical derivation as presented in Lemma 1, we readily derive:

$$E_{q}(z)=z+ic_{2}z^{2}-c_{3}z^{3}-ic_{4}z^{4}+c_{5}z^{5}+\cdots ,$$

where $c_{n}$ for n=2,3,4,5 is given by equations (18), (19), (20) and (21) respectively. Consequently, for the function $E_{q}(z)$ and n=1,2,3, we find:

$$|T_{2}(n)|=c_{n}^{2}+c_{n+1}^{2},$$

demonstrating that $E_{q}(z)$ serves as an extremal function for the inequalities (28), (29), and (30). □

For $q\to 1^{-}$, we have the following known result.

Corollary 7 [18]*If*$f\in S^{⁎}$*, then*

$$|T_{2}(1)|=|a_{1}^{2}-a_{2}^{2}|\leq 5,|T_{2}(2)|=|a_{2}^{2}-a_{3}^{2}|\leq 13,|T_{2}(3)|=|a_{3}^{2}-a_{4}^{2}|\leq 25.$$

Theorem 7 *If*$f\in S_{q}^{⁎}$*, then the sharp inequality*

$$|T_{3}(1)|=|1-2a_{2}^{2}+2a_{2}^{2}a_{3}-a_{3}^{2}|\leq 1+8q_{1}^{2}-\frac{4q_{1}^{2}}{{\left[2\right]}_{q}^{2}}+\frac{8q_{1}^{3}}{{\left[2\right]}_{q}}+12q_{1}^{4}$$

*holds.*

Proof Let $f\in S_{q}^{⁎}$ be given by equation (1), then

$$T_{3}(1)=\left|\begin{array}{ccc} 1 & a_{2} & a_{3}\\ a_{2} & 1 & a_{2}\\ a_{3} & a_{2} & 1 \end{array}\right|=1-2a_{2}^{2}+2a_{2}^{2}a_{3}-a_{3}^{2}.$$

Taking absolute and applying the triangle inequality, we can write

$$|T_{3}(1)|\leq 1+2|a_{2}{|}^{2}+|a_{3}||a_{3}-2a_{2}^{2}|.$$

Using Lemma 1 and applying Theorem 1, we obtain

$$|T_{3}(1)|\leq 1+8q_{1}^{2}+\left(\frac{2q_{1}}{{\left[2\right]}_{q}}+2q_{1}^{2}\right)\left(\frac{2q_{1}}{{\left[2\right]}_{q}}\max\left\{1,\left|3q_{1}{\left[2\right]}_{q}-1\right|\right\}\right)=1+8q_{1}^{2}-\frac{4q_{1}^{2}}{{\left[2\right]}_{q}^{2}}+\frac{8q_{1}^{3}}{{\left[2\right]}_{q}}+12q_{1}^{4}.$$

For the function $E_{q}(z)$ defined in (31), we find:

$$|T_{3}(1)|=1+2c_{2}^{2}+2c_{2}^{2}c_{3}-c_{3}^{2}=1+8q_{1}^{2}-\frac{4q_{1}^{2}}{{\left[2\right]}_{q}^{2}}+\frac{8q_{1}^{3}}{{\left[2\right]}_{q}}+12q_{1}^{4},$$

which validates the sharpness of the inequality. □

For $q\to 1^{-}$, we have the following known result.

Corollary 8 [18]*If*$f\in S^{⁎}$*, then*

$$|T_{3}(1)|=|1-2a_{2}^{2}+2a_{2}^{2}a_{3}-a_{3}^{2}|\leq 24.$$

Theorem 8 *If*$f\in S_{q}^{⁎}$*, then*

$$|T_{3}(2)|=|(a_{2}-a_{4})(a_{2}^{2}-2a_{3}^{2}+a_{2}a_{4})|\leq B_{3}(q),$$

*where*

$$B_{3}(q)=8\left(q_{1}+\frac{q_{1}}{{\left[3\right]}_{q}}+\frac{2q_{1}^{2}}{{\left[2\right]}_{q}}+\frac{2q_{1}^{3}}{3}\right)\left(q_{1}^{2}+\frac{4q_{1}^{4}}{3}+\frac{2q_{1}^{2}}{{\left[2\right]}_{q}^{2}}+\frac{2q_{1}^{3}}{{\left[2\right]}_{q}}-\frac{q_{1}^{2}}{{\left[3\right]}_{q}}\right).$$

Proof Let $f\in S_{q}^{⁎}$ be given by equation (1), then

$$T_{3}(2)=\left|\begin{array}{ccc} a_{2} & a_{3} & a_{4}\\ a_{3} & a_{2} & a_{3}\\ a_{4} & a_{3} & a_{2} \end{array}\right|=(a_{2}-a_{4})(a_{2}^{2}-2a_{3}^{2}+a_{2}a_{4}).$$

Taking absolute and applying the triangle inequality, we can write

(32) $$|T_{3}(2)|=|a_{2}-a_{4}||a_{2}^{2}-2a_{3}^{2}+a_{2}a_{4}|.$$

Taking absolute value and using Lemma 1 we get

(33) $$|a_{2}-a_{4}|\leq |a_{2}|+|a_{4}|\leq 2q_{1}+\frac{2q_{1}}{{\left[3\right]}_{q}}+\frac{4q_{1}^{2}}{{\left[2\right]}_{q}}+\frac{4q_{1}^{3}}{3}.$$

Using equations (10), (11) and (12), we can write

$$a_{2}^{2}-2a_{3}^{2}+a_{2}a_{4}=q_{1}^{2}p_{1}^{2}-\frac{q_{1}^{4}p_{1}^{4}}{3}-\frac{2q_{1}^{2}p_{2}^{2}}{{\left[2\right]}_{q}^{2}}+\frac{q_{1}^{2}p_{1}}{{\left[3\right]}_{q}}\left(p_{3}-\frac{q_{1}{\left[3\right]}_{q}}{{\left[2\right]}_{q}}p_{1}p_{2}\right).$$

Taking absolute value, applying the triangle inequality and using Lemma 2, Lemma 4 we have

(34) $$|a_{2}^{2}-2a_{3}^{2}+a_{2}a_{4}|\leq q_{1}^{2}|p_{1}{|}^{2}+\frac{q_{1}^{4}}{3}|p_{1}{|}^{4}+\frac{2q_{1}^{2}}{{\left[2\right]}_{q}^{2}}|p_{2}{|}^{2}+\frac{q_{1}^{2}}{{\left[3\right]}_{q}}|p_{1}|\left|p_{3}-\frac{q_{1}{\left[3\right]}_{q}}{{\left[2\right]}_{q}}p_{1}p_{2}\right|\leq 4q_{1}^{2}+\frac{16q_{1}^{4}}{3}+\frac{8q_{1}^{2}}{{\left[2\right]}_{q}^{2}}+\frac{4q_{1}^{2}}{{\left[3\right]}_{q}}\max\{1,\left|\frac{2q_{1}{\left[3\right]}_{q}}{{\left[2\right]}_{q}}-1\right|\}\leq 4q_{1}^{2}+\frac{16q_{1}^{4}}{3}+\frac{8q_{1}^{2}}{{\left[2\right]}_{q}^{2}}+\frac{8q_{1}^{3}}{{\left[2\right]}_{q}}-\frac{4q_{1}^{2}}{{\left[3\right]}_{q}}.$$

Using inequalities (33) and (34) in (32), we get the required inequality.

For the function $E_{q}(z)$ defined in (31), we find:

$$|T_{3}(2)|=|c_{2}+c_{4}||c_{2}^{2}+2c_{3}^{2}-c_{2}c_{4}|=B_{3}(q),$$

which establishes the sharpness of the inequality. □

For $q\to 1^{-}$, we have the following known result.

Corollary 9 [18]*If*$f\in S^{⁎}$*, then*

$$|T_{3}(2)|=|(a_{2}-a_{4})(a_{2}^{2}-2a_{3}^{2}+a_{2}a_{4})|\leq 84.$$

## 5 Conclusions

In this present article, we have successfully obtained the sharp upper bounds for the second order Hankel determinants $\left|H_{2}(1)|,|H_{2}(2)\right|$ and related functionals $\left|a_{2}a_{3}-a_{4}\right|$, $\left|a_{2}a_{5}-a_{3}a_{4}\right|$ for *q*-starlike functions. We also find an upper bound for the third order Hankel determinant $\left|H_{3}(1)\right|$ along with the sharp upper bounds for Toeplitz determinant $\left|T_{m}(n)\right|$, where (m,n)∈{(2,2),(2,3),(3,1),(3,2)} are attained. The results obtained generalize the known results for univalent starlike functions to the larger class of *q*-starlike functions and also provide the foundation for establishing precise upper bounds for Hankel and Toeplitz determinants of higher orders. The extremal functions identified through these inequalities have the potential to enhance previously derived inequalities for various subclasses of *q*-starlike functions.

## CRediT authorship contribution statement

**Ihtesham Gul:** Writing – original draft, Methodology, Formal analysis, Conceptualization. **Sa'ud Al-Sa'di:** Writing – original draft, Validation, Investigation, Formal analysis. **Saqib Hussain:** Validation, Supervision, Conceptualization. **Saima Noor:** Validation, Funding acquisition, Formal analysis.

## Declaration of Competing Interest

The authors declare that they have no known competing financial interests or personal relationships that could have appeared to influence the work reported in this paper.

## Data Availability

No data was used for the research described in the article.
